# Sex difference in oral frailty Among older hospitalized cancer patients: a cross-sectional survey in taizhou, China

**DOI:** 10.3389/fdmed.2026.1899112

**Published:** 2026-09-30

**Authors:** Wei-dan Wu, Shanshan Lin, Tao-Hsin Tung, Yu-Pei Yang

**Affiliations:** 1 Department of Gastroenterology, Taizhou Hospital of Zhejiang Province, Wenzhou Medical University, Linhai, Zhejiang, China; 2 Evidence-based Medicine Center, Taizhou Hospital of Zhejiang Province Affiliated to Wenzhou Medical University, Linhai, Zhejiang, China; 3 Department of Hematology, Taizhou Hospital of Zhejiang Province Affiliated to Wenzhou Medical University, Linhai, Zhejiang, China

**Keywords:** cancer, influencing factor, older, oral frailty, proportion at high risk

## Abstract

**Background:**

Oral frailty is an associated factor for frailty and may increase the likelihood of transitioning from pre-frailty to frailty in older patients. Therefore, this study analyzed factors associated with oral frailty in older hospitalized patients with cancer in Taizhou, China.

**Methods:**

We included 190 older patients with cancer who were hospitalized from April to June 2024 were selected by convenience sampling in a tertiary hospital in Taizhou. A general information questionnaire and the Mini Nutritional Assessment - Short Form, Oral Health Assessment Tool and Oral Frailty Index-8 were used for the investigation. Multivariable logistic regression was used to identify factors associated with oral frailty.

**Results:**

190 older in-patients with cancer were included in the study, comprising 111 men and 79 women. The proportion at high risk of oral frailty was 54.7% (men: 57.7% vs. women: 50.6%, *p* = 0.338). Based on the multivariable logistic regression, patients who with oral frailty had significantly higher odds of denture (OR = 7.710, 95% CI: 2.932–20.275, *p* < 0.001) and malnutrition (OR = 2.607, 95% CI: 1.197–5.678, *p* = 0.016). The disparity was also found in the sex-stratified subgroup analyses. In women cancer patients, denture (OR = 6.417, 95% CI: 1.717–23.986, *p* = 0.006) was the significant factor related to oral frailty. In men cancer patients, denture (OR = 15.858, 95% CI: 3.227–77.929, *p* < 0.001), xerostomia (OR = 12.725, 95% CI: 1.607–100.763, *p* = 0.016), malnutrition (OR = 2.972, 95% CI: 1.010–8.743, *p* = 0.048), and poor oral health (OR = 5.279, 95% CI: 1.009–27.632, *p* = 0.049) were associated with oral frailty.

**Conclusions:**

Oral frailty was common among older cancer patients. Denture and malnutrition may help identify patients at increased likelihood of oral frailty. Therefore, early identify and appropriate treatment were required given the differences in the associated factors to oral frailty among men and women older cancer patients.

## Background

Cancer remains a leading cause of global mortality and a major impediment to improvements in population life expectancy (1, 2). The World Health Organization has recognized cancer as one of the top two contributors to premature death among individuals aged under 70 years across 183 countries (3). Driven by global population aging, the societal and public health burden of cancer continues to escalate worldwide (4). In China, the aging population has surpassed 300 million, placing the nation in a stage of deep population aging (5). Older adults bear a disproportionate cancer burden, accounting for 60.6% of all new cancer diagnoses and 76.7% of cancer-related deaths nationally. Globally, older Chinese individuals contribute 22.6% of new cancer cases and 27.9% of cancer deaths in the older population (5, 6). The rising morbidity and mortality attributable to cancer in aging populations represents an urgent public health challenge (7). Frailty is a geriatric syndrome characterized by diminished physiological reserve and increased vulnerability to stressors, and is strongly predictive of functional decline, care dependency, and adverse clinical outcomes in older adults (8, 9). In aging societies such as China, frailty exacerbates vulnerability to acute physiological stress and compromises the tolerability and efficacy of standard cancer treatment, creating substantial clinical barriers to optimal oncological care in older patients (10).

Emerging evidence supports a robust association between oral health status and frailty progression, with tooth loss and periodontal disease identified as independent risk factors for incident frailty (11). The construct of oral frailty, defined as progressive deterioration in oral physiological function, was formally proposed in 2015 to characterize early oral functional impairment in older adults (12). The pre-frailty stage typically begins with reduced social engagement and limited oral health literacy, followed by subtle oral functional deficits, including decreased tongue mobility, chewing impairment, and food retention during mastication (13). Notably, oral frailty precedes the development of physical frailty and represents a reversible preclinical stage before the establishment of irreversible geriatric vulnerability, rendering it a critical intervention window for preserving physical function in older populations (13). Accumulating data further validate oral frailty as a significant correlate of physical frailty, functional dependence, and all-cause mortality in older adults (14).

In response to the growing burden of age-related oral dysfunction, China launched the National Healthy Mouth Action Program (2019–2025), prioritizing oral health promotion and disease prevention among older adults and emphasizing the systemic link between oral and general health (15). Oral frailty is closely linked to major adverse geriatric outcomes, including functional disability, reduced quality of life, recurrent hospitalization, falls, and premature mortality (16, 17). Previous research has demonstrated that structured in-hospital oral care interventions may improve survival outcomes and functional status in vulnerable older patients (18). Epidemiological studies report the prevalence of oral frailty among hospitalized older adults to range from 30.4% to 64.3% (19, 20). Given its subclinical and underrecognized nature, targeted preservation of oral function is particularly critical for older patients with cancer, as oral frailty may adversely affect treatment tolerance, functional recovery, and long-term survival. Early identification of associated risk factors and timely intervention are therefore essential to mitigate frailty-related adverse outcomes in this high-risk population (21).

Oral frailty is tightly coupled with physical frailty trajectories and accelerates the transition from pre-frailty to overt frailty in older adults. Compelling evidence indicates that impaired oral health exacerbates frailty risk and negatively modulates clinical prognosis in hospitalized older populations (22, 23). Against this background, the present study aimed to characterize the prevalence of oral frailty and explore its associated factors among hospitalized older patients with cancer.

## Methods

### Study design and data collection

We conducted a cross-sectional study and recruited participants by convenience sampling. An anonymous hospital-based survey was conducted from April to June 2024 via a questionnaire in Taizhou Hospital of Zhejiang Province. Researchers who had received standardized training introduced the purpose and significance of the survey to participants. Upon obtaining oral consent, paper questionnaires were distributed on-site, with face-to-face guidance provided to the older cancer participants for completion. We did not ask for a separate written informed consent because of anonymity. Questionnaires were collected and immediately checked. For patients facing challenges, such as low literacy or presbyopia, researchers employed consistent language in reading and explaining questions while assisting them in providing responses. The study was approved by the Ethics Committee of Taizhou Hospital of Zhejiang Province (approval number: K20240444) in China. All programs were carried out according to the standards of our ethics committee and adhered to the tenets of the Declaration of Helsinki. Oral rather than written consent was used and the information of respondents was kept anonymous. All respondents' information was anonymous.

Inclusion criteria were 1) aged ≥60 years, 2) pathological or imaging diagnosis of malignant tumors, and 3) ability to cooperate with the completion of research. The exclusion criteria were: 1) previous serious mental illness; 2) and those with severe heart, brain, kidney and other organ dysfunction or in the acute phase of the disease; and 3) patients who were unable to fill out the questionnaire or had difficulties with communication. The required sample size was determined *a priori* using G*Power version 3.1 (University of Kiel, Germany) (24). Prospective sample size estimation for two-sided binary logistic regression was performed in G*Power 3.1.9.2. We set *α* = 0.05, powe*r* = 0.85, odds ratio=2.00, baseline event probability = 0.70, and R^2^of additional covariates = 0.30. The minimal sample size required was 149. To compensate for a 20% incomplete data rate, we planned to recruit 186 participants in total. 200 questionnaires were distributed, resulting in 190 effectively recovered responses and an effective recovery rate of 95%. Figure 1 illustrated the flowchart depicting the process of patient screening.

**Figure 1:**
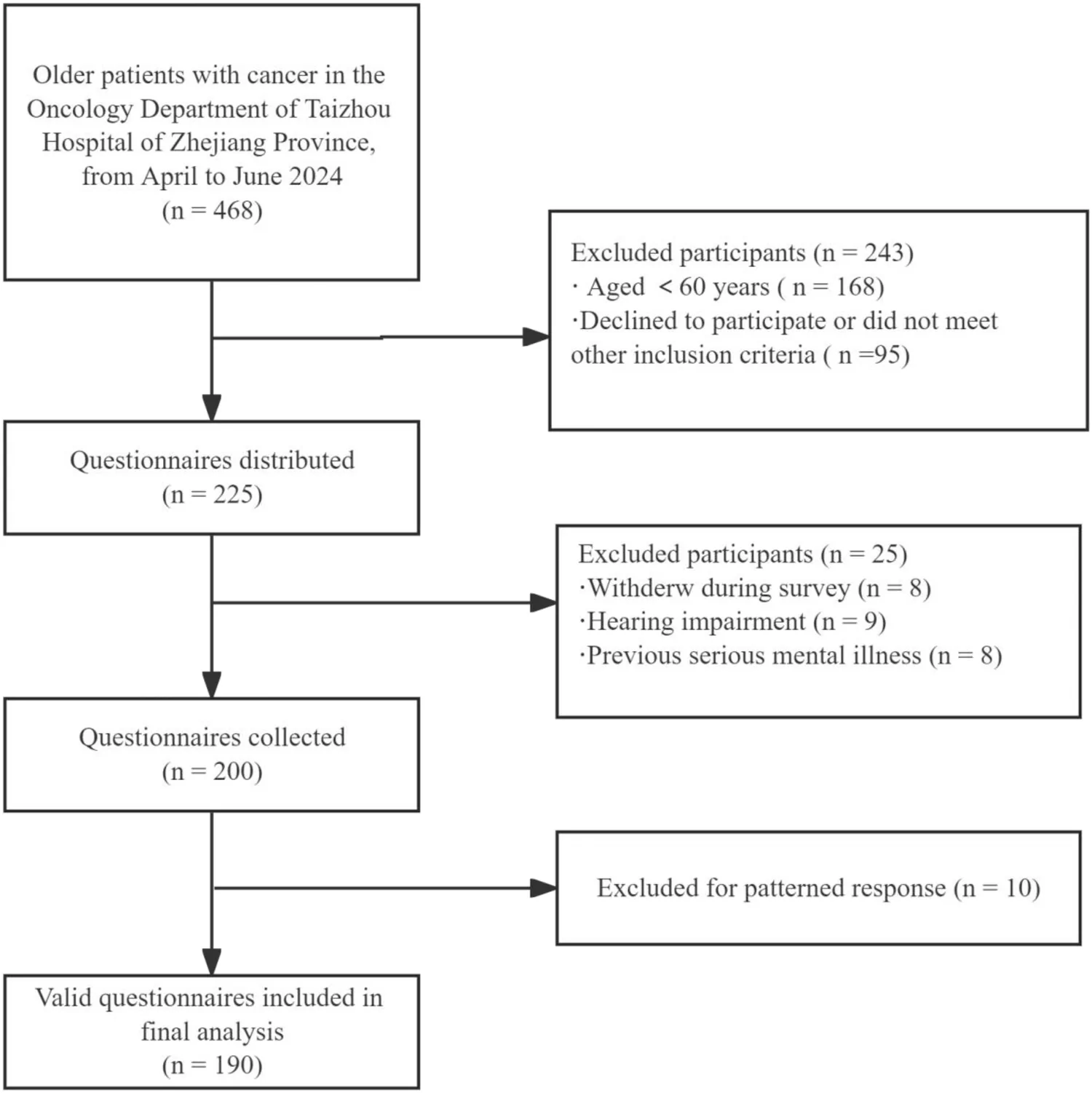
Flowchart of patients' enrollment.

### Measurements

Data were collected using a structured questionnaire comprising several sections: demographic characteristics, clinical characteristics, the Mini Nutritional Assessment - Short Form (MNA-SF), Oral Health Assessment Tool (OHAT), and Oral Frailty Index-8 (OFI-8). Figure 2 depicted the conceptual framework for studying the outcome variable.

**Figure 2:**
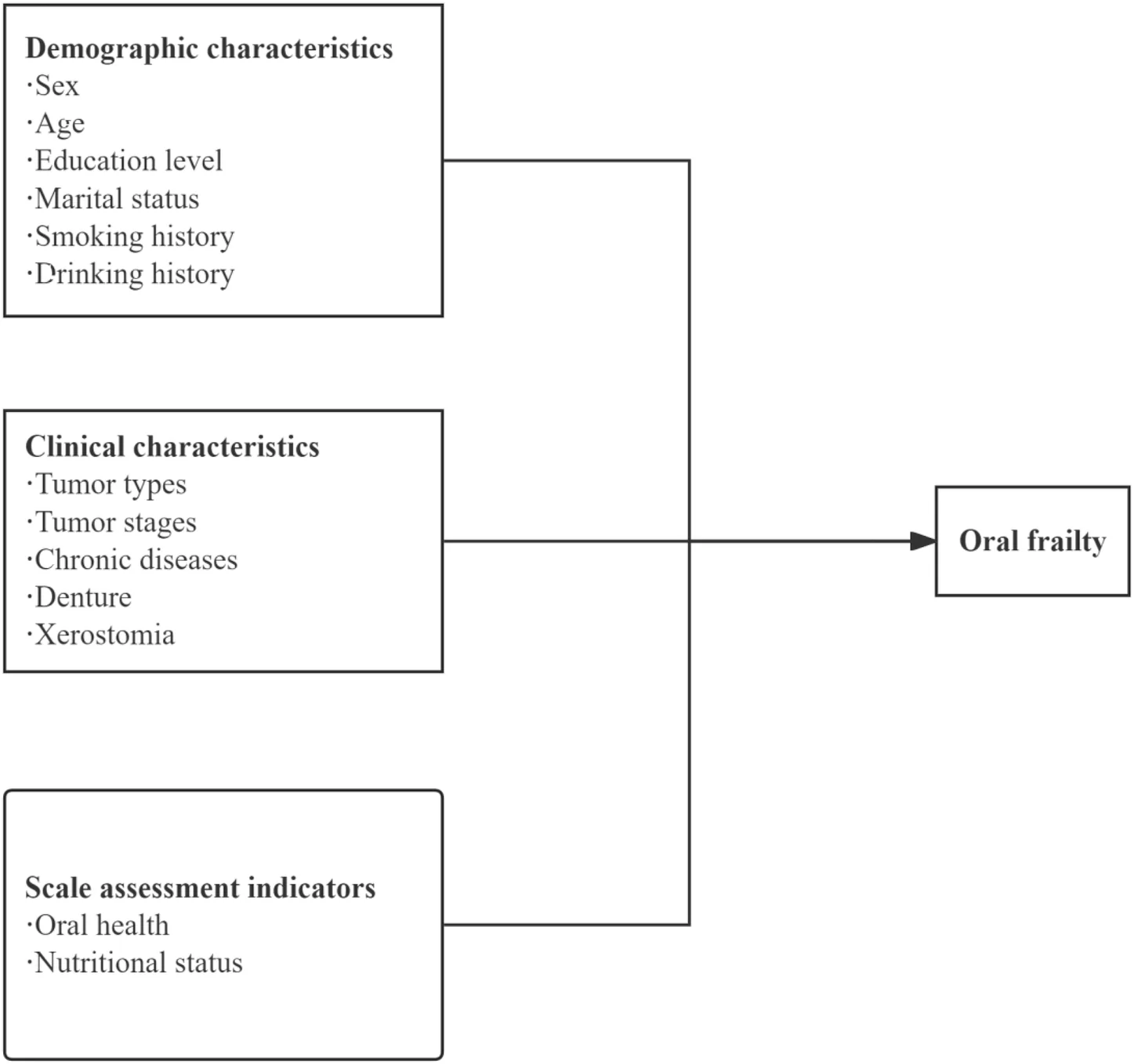
Conceptual framework for studying the outcome variable.

#### Demographic and clinical characteristics

This included sex, age, education level, marital status, smoking history, alcohol history,

tumor types, tumor stages, chronic diseases, denture and xerostomia.

#### MNA-SF

The MNA-SF is a short form of the MNA designed for the older. It contains six questions selected from the MNA, which assess recent weight loss, changes in appetite, mobility, psychological stress, neuropsychological problems, and body mass index. Scores for each question range from 0 to 3, with a maximum score of 14. A score of ≥11 indicates normal nutrition, and a score of <11 indicates malnutrition according to previous research (25). Moreover, the MNA-SF is easy to use and can be utilized by non-nutritional professionals with routine training (26). The Cronbach's ɑ coefficient in this study was 0.712.

#### OHAT

As demonstrated by Chalmers et al., the OHAT is a practical tool for measuring overall oral health (27). It has been translated into several versions and is widely used (28, 29). The items assessed by the OHAT include the lips, tongue, gums and tissues, saliva, real teeth, dentures, oral cleanliness, and dental pain. The instrument could be used with people with cognitive impairment as it does not require them to express themselves. Each item was scored between 0 (healthy) and 2 (unhealthy). Two categories are possible based on the overall score, which ranges from 0 to 16: “healthy mouth” (<3) and “unhealthy mouth” (≥3). The Chinese version of the OHAT has a Cronbach's value of 0.710 and a retest reliability of 0.811, which can be used to assess the oral health status of Chinese older adults (30). The Cronbach's ɑ coefficient in this study was 0.735.

#### OFI-8

The presence or absence of oral frailty likelihood was assessed on the basis of eight items, which is a modification of the method described by Tanaka et al. (31). The OFI-8 is an 8-item screening questionnaire that integrates oral health-related behaviors and concepts of frailty. The OFI-8 doubles the score of the three higher-priority items for the most important elements of oral frailty (“tooth loss,” “subjective chewing difficulties,” and “subjective swallowing difficulties”). The OFI-8 total score ranges from 0 to 11 points, higher scores indicate poorer oral health. The higher the OFI-8 score, the higher the risk of oral frailty; a score of 0–2 points indicate low risk; a score of 3 points indicate moderate risk; and a score of 4–11 points indicate high risk. In this study, an OFI-8 score ≥4 was defined as oral frailty and high risk (32). The Cronbach's ɑ coefficient in this study was 0.731.

### Statistical analysis

The results were presented as percentages (%) for categorical variables. Oral frailty, defined as an OFI-8 score ≥4, was designated as the dependent variable. In the univariable analysis, variables with a *p*-value < 0.2 were retained and subsequently entered into the multivariable regression model. This liberal threshold was applied to avoid eliminating potential meaningful confounding factors with marginal statistical associations, which is a well-recognized strategy in observational clinical research to preserve statistical power and minimize variable selection bias (33). All clinically meaningful covariates identified based on biological plausibility and literature review were also entered into the multivariable logistic regression model to estimate adjusted odds ratios (OR) and 95% confidence intervals (CI). The possible interactions were also explored. Additionally, we further performed sex-stratified subgroup analyses by fitting separate multivariable models for women and men. A two-tailed *P*-value < 0.05 was considered statistically significant. All statistical analyses were performed using IBM SPSS Statistics for Windows (version 29.0; IBM Corporation, Armonk, NY, USA).

## Results

A total of 190 older patients hospitalized for cancer were enrolled in this study. As shown in Table 1, 111 (58.4%) were men and 79 (41.6%) women. Of these, 96 (50.5%) were aged ≥70 years and 94 (49.5%) aged 60- 69 years. The majority had a primary school degree or below (85.8%), and 93.7% of participants were married. In addition, 28.4% patients with cancer had chronic diseases. Among all patients, 27.9% had denture, 34.7% experienced xerostomia, and 12.1% had malnutrition. Approximately half (57.9%) of the patients had an unhealthy mouth. The proportion of participants at high risk of oral frailty was 54.7%.

**Table 1:** The demographic characteristics of older cancer hospitalized patients (*n* = 190).

Variables	Categories	*n* (%)
Categorical variables		
Sex	Men	111 (58.4)
	Women	79 (41.6)
Age (years)	60–69	94 (49.5)
	≥70	96 (50.5)
Education level	Primary and below	163 (85.8)
	Junior high school and above	27 (14.2)
Marital status	Married	178 (93.7)
	Others	12 (6.3)
Smoking history	Yes	16 (8.4)
	No	174 (91.6)
Drinking history	Yes	5 (2.6)
	No	185 (97.4)
Tumor types	Gastrointestinal tract tumors	72 (37.9)
	Lung cancer	23 (12.1)
	Hepatobiliary, splenic, and pancreatic tumors	32 (16.8)
	Blood system tumors	36 (18.9)
	Thyroid and breast neoplasms	8 (4.3)
	Gynecological cancer	7 (3.7)
	Others	12 (6.3)
Tumor stages	Ⅰ	19 (10.0)
	Ⅱ	25 (13.2)
	Ⅲ	29 (15.2)
	Ⅳ	53 (27.9)
	Unknown	64 (33.7)
Chronic diseases	Yes	54 (28.4)
	No	136 (71.6)
Denture	Yes	53 (27.9)
	No	137 (72.1)
Xerostomia	Yes	66 (34.7)
	No	124 (65.3)
Oral frailty	yes	104 (54.7)
	No	86 (45.3)
Nutritional Status	Malnutrition	23 (12.1)
	Normal	167 (87.9)
Oral Health	Healthy	80 (42.1)
	Unhealthy	110 (57.9)

As shown in Table 2, patients with denture (86.8% vs. 42.3%, *p* < 0.001), xerostomia (77.3% vs. 42.7%, *p* < 0.001), malnutrition (71.3% vs. 42.7%, *p* < 0.001), and poor oral health (70.0% vs. 33.3%, *p* < 0.001) had higher prevalence of oral frailty. All variables were coded as dummy variables, with specific variable coding details shown in Table 3. The multivariable logistic regression analysis revealed that denture (OR = 7.710, 95% CI: 2.932–20.275, *p* < 0.001), and malnutrition (OR = 2.607, 95% CI: 1.197–5.678, *p* = 0.016) were positively associated with oral frailty in hospitalized older patients with cancer (Table 4).

**Table 2:** Univariable analysis of the factor to oral frailty in older cancer hospitalized patients (*n* = 190).

Variables	Categories	Non-oral frailty (*n* = 86)	Oral frailty (*n* = 104)	Crude *OR*	95% CI	*P* value
		*n* (%)	*n* (%)			
Sex	Men	47 (42.3)	64 (57.7)	1.328	(0.743–2.371)	0.338
	Women	39 (49.4)	40 (50.6)	-	-	-
Age (years)	60–69	46 (48.9)	48 (51.1)	1.342	(0.757–2.379)	0.315
	≥70	40 (41.7)	56 (58.3)	-	-	-
Education level	Primary and below	74 (45.4)	89 (54.6)	0.962	(0.424–2.183)	0.926
	Junior high school and above	12 (44.4)	15 (55.6)	-	-	-
Marital status	Married	32 (18.0)	146 (82.0)	0.585	(0.170–2.014)	0.396
	Others	2 (16.7)	10 (83.3)	-	-	-
Smoking history	Yes	9 (56.3)	7 (43.7)	0.617	(0.222–1.733)	0.360
	No	77 (44.3)	97 (55.7)	-	-	-
Drinking history	Yes	3 (60.0)	2 (40.0)	0.542	(0.089–3.323)	0.508
	No	83 (44.9)	102 (55.1)	-	-	-
Tumor types	Gastrointestinal tract tumors	30 (41.7)	42 (58.3)	-	-	-
	Lung cancer	9 (39.1)	14 (60.9)	1.111	(0.426–2.901)	0.830
	Hepatobiliary, splenic, and pancreatic tumors	20 (62.5)	12 (37.5)	0.429	(0.182–1.008)	0.052
	Blood system tumors	16 (44.4)	20 (55.6)	0.893	(0.398–2.002)	0.783
	Thyroid and breast neoplasms	2 (25.0)	6 (75.0)	2.143	(0.112–2.571)	0.370
	Gynecological cancer	4 (57.1)	3 (42.9)	0.536	(0.112–2.571)	0.435
	Others	5 (41.7)	7 (58.3)	1.000	(0.290–3.454)	1.000
Tumor stages	Ⅰ	8 (42.1)	11 (57.9)	-	-	-
	Ⅱ	14 (56.0)	11 (44.0)	0.571	(0.171–1.908)	0.363
	Ⅲ	11 (37.9)	18 (62.1)	1.190	(0.366–3.872)	0.773
	Ⅳ	25 (47.2)	28 (52.8)	0.815	(0.283–2.347)	0.704
	Unknown	28 (43.8)	36 (56.2)	0.935	(0.332–2.635)	0.899
Chronic diseases	Yes	27 (50.0)	27 (50.0)	0.766	(0.407–1.442)	0.409
	No	59 (43.4)	77 (56.6)	-	-	-
Denture	Yes	7 (13.2)	46 (86.8)	8.951	(3.771–21.245)	<0.001
	No	79 (57.7)	58 (42.3)	-	-	-
Xerostomia	Yes	15 (22.7)	51 (77.3)	4.555	(2.315–8.961)	<0.001
	No	71 (57.3)	53 (42.7)	-	-	-
Nutritional Status	Malnutrition	23 (28.7)	57 (71.3)	3.322	(1.798–6.119)	<0.001
	Normal	63 (57.3)	47 (42.7)	-	-	-
Oral health	Healthy	53 (66.7)	27 (33.3)	4.580	(2.471–8.491)	<0.001
	Unhealthy	33 (30.0)	77 (70.0)	-	-	-

**Table 3:** The coding and reference categories of all variables.

Variables	Coding and Reference categories
Age	0 = 60–69 yrs; 1=≥70 yrs
Sex	0 = women; 1 = men
Denture	0 = no; 1 = yes
Xerostomiah	0 = no; 1 = yes
Nutritional status	0 = normal; 1 = malnutrition
Oral health	0 = healthy; 1 = unhealthy

**Table 4:** Multivariable logistic regression of the oral frailty in older cancer hospitalized patients (*n* = 190).

Variables	*β*	S.E.	Wald	*P* value	OR	95%CI
Constant	−1.617	0.476	11.550	<0.001	-	-
Age (≥70 yrs vs. 60–69 yrs)	−0.457	0.375	1.488	0.223	0.633	(0.304–1.320)
Sex (men vs. women)	0.551	0.370	2.220	0.136	1.734	(0.841–3.578)
Denture (yes vs. no)	2.043	0.493	17.143	<0.001	7.710	(2.932–20.275)
Xerostomiah (yes vs. no)	1.387	0.748	3.438	0.064	4.001	(0.924–17.326)
Nutritional status (malnutrition vs. normal)	0.958	0.397	5.821	0.016	2.607	(1.197–5.678)
Oral health (unhealthy vs. healthy)	0.599	0.584	1.051	0.305	1.819	(0.579–5.714)
Oral health×Xerostomiah	−0.013	0.165	0.006	0.938	0.987	(0.714–1.364)
Oral health×Nutritional Status	0.003	0.014	0.048	0.826	1.003	(0.976–1.031)
*P* value for Hosmer-Lemeshow Test =0.080, Omnibus test: χ=66.344, *P* < 0.001, Nagelkerke R^2^ = 0.394, C-statistic=0.771.						

Sex-stratified analyses were further performed to examine the associations of potential associated factors with oral frailty (Table 5). Among women patients, denture was significantly associated with oral frailty (OR = 6.417, 95% CI: 1.717–23.986, *p* = 0.006). Among men patients, denture (OR = 15.858, 95% CI: 3.227–77.929, *p* < 0.001), xerostomia (OR = 12.725, 95% CI: 1.607–100.763, *p* = 0.016), malnutrition (OR = 2.972, 95% CI: 1.010–8.743, *p* = 0.048), and poor oral health (OR = 5.279, 95% CI: 1.009–27.632, *p* = 0.049) were associated with oral frailty.

**Table 5:** Sex-stratified analysis of multivariable logistic regression of the oral frailty in older cancer hospitalized patients (*n* = 190).

Variables	Women (*n* = 79)	Men (*n* = 111)		
	Adjusted OR (95%CI)	*P* value	Adjusted OR (95%CI)	*P* value
Age (≥70 yrs vs. 60–69 yrs)	0.871 (0.299–2.542)	0.801	0.410 (0.134–1.250)	0.117
Denture (yes vs. no)	6.417 (1.717–23.986)	0.006	15.858 (3.227–77.929)	<0.001
Xerostomiah (yes vs. no)	1.030 (0.086–12.283)	0.981	12.725 (1.607–100.763)	0.016
Nutritional status (malnutrition vs. normal)	3.037 (0.824–11.196)	0.095	2.972 (1.010–8.743)	0.048
Oral health (unhealthy vs. healthy)	0.509 (0.075–3.433)	0.488	5.279 (1.009–27.632)	0.049
Oral health×Xerostomiah	1.222 (0.736–2.031)	0.438	0.857 (0.522–1.407)	0.542
Oral health×Nutritional Status	1.011 (0.971–1.052)	0.596	0.991 (0.952–1.032)	0.677

Women- *P* value for Hosmer-Lemeshow Test =0.700, Omnibus test: χ=22.724, *P* = 0.002, Nagelkerke R^2^ = 0.333, C-statistic=0.827.

Men- *P* value for Hosmer-Lemeshow Test =0.736, Omnibus test: χ=51.599, *P* < 0.001, Nagelkerke R^2^ = 0.600, C-statistic=0.694.

## Discussion

### Clinical implications

This study investigated the sex differences of associated factors for oral frailty in older hospitalized cancer patients. The results revealed distinct associations between clinical factors and oral frailty across different sex groups.

In this study, the proportion of participants at high risk of oral frailty (54.7%) was slightly lower than the results of Li et al. (57.6%) and Wang et al. (57.7%) (34, 35). However, it was relatively higher than the prevalence among community-dwelling older adults in China (33.8%) and those residing in Chinese aged care facilities (31.0%) (36, 37). Several factors may account for these disparities. First, the discrepancies might be due to higher prevalence of physical frailty, sarcopenia, and malnutrition among hospitalized patients (38). Second, cancer treatment may have negative impacts on oral health (39). Radiotherapy and chemotherapy are both likely to cause xerostomia mouth, oral mucositis, and changes in taste, continuously damaging the chewing and swallowing functions of the mouth, and increasing the risk of oral frailty (40). Third, subclinical oral dysfunction often predates cancer diagnosis. However, post-hospitalization symptoms such as nausea, anorexia, and restricted mobility further compromise patients' capacity for routine oral self-care. When superimposed on pre-existing oral conditions and tumor-induced physiological stress, these hospital-acquired barriers collectively accelerate the manifestation of overt oral frailty (41). Finally, variations in tumor type distribution, the proportion of patients receiving radiotherapy, and the heterogeneity of treatment regimens may also contribute to the observed fluctuations in prevalence (42, 43).

Denture emerged as an independent associated factor for oral frailty across all sex groups. The present findings are consistent with a previously published meta-analysis, which also identified denture wearing as a significant contributor to oral frailty (44). Serving as artificial replacements for natural teeth, dentures impair masticatory function, restrict dietary intake and reduce patients' overall quality of life (45, 46). Among removable denture wearers, ill fit between prostheses and alveolar bone loosens the underlying mucosal tissues. Chronic mechanical friction and persistent mucosal deformation create a favorable microenvironment for the colonization and overgrowth of oral pathogenic fungi, increasing the risk of denture-related fungal infections (47). Many older adults who wear dentures struggle with insufficient saliva production, irregular oral hygiene practices and trapped food residues. These problems exacerbate prosthetic complications and further aggravate oral frailty (48). Poor denture hygiene and accumulated food debris commonly trigger denture stomatitis and recurrent oral ulcers, which accelerate the progression of oral frailty (49).

In this study, malnutrition was correlated with oral frailty in older cancer patients, sex-stratified subgroup stratification confirmed a positive correlation only in men rather than women. Research has demonstrated that certain chemotherapy drugs can be secreted into the oral cavity through saliva or capillary networks, where they directly interact with taste receptors, resulting in a bitter or metallic taste (33). When patients experience taste alterations, their appetite is often diminished, leading to malnutrition (50). Malnutrition, in turn, compromises the health of the oral mucosa and can lead to decreased oral muscle strength, affecting chewing and swallowing functions, and further increasing the risk of oral frailty (51). One study had verified that an inverse association exists between elevated dietary protein intake and the prevalence of comprehensive physical frailty (52). Deficient intake of protein, vitamins and trace elements markedly weakens the regenerative capacity of oral mucosa and slows tissue healing, making patients prone to recurrent oral ulcers and gingival recession (53). Moreover, progressive declines in occlusal force and swallowing function not only serve as triggers but also perpetuate the aggravation of oral frailty, ultimately establishing a mutually reinforcing cycle between malnutrition and oral functional impairment (54). The higher prevalence of malnutrition in men is attributed to their significantly higher prevalence of malnutrition compared with women (55).

This study further showed the association between poor oral health and oral frailty in older men cancer patients. Men are more likely to experience higher rates of periodontal disease, oral cancer and dental trauma (35, 56). Men are generally less health-conscious than women, tending to neglect oral hygiene and underestimate its systemic relevance (57). Poor oral hygiene disturbs the balance of oral microbiota. Persistent oral discomfort drives patients to stick to soft diets, causing disuse atrophy of facial and jaw muscles (58). Nutritionally, disease progression and chemotherapy-induced nausea exacerbate the impact of poor oral status on nutritional intake (59). Biologically, elevated baseline inflammation from malignancy or anticancer therapies may exacerbate the effects of oral health deterioration on muscle function and metabolism, increasing frailty risk (60). Psychosocially, oral health–related depression, embarrassment, and activity restrictions may promote social isolation, sedentary behavior, and cognitive decline (61). Moreover, cancer-specific toxicities—including mucositis, xerostomia, taste alterations, and infection—can worsen oral health, establishing a bidirectional cycle in which frailty further compromises oral hygiene maintenance (62).

In addition, this study revealed that xerostomia was associated with oral frailty in older man patients with cancer. Verma et al.'s study indicated that man cancer patients had a higher proportion of xerostomia symptoms than women (63). Xerostomia in older men cancer patients arises from age-related salivary gland atrophy, irreversible salivary gland damage induced by radiotherapy and chemotherapy, anticholinergic side effects of analgesics and medications for chronic comorbidities, as well as dehydration caused by tumor-induced metabolic consumption (64, 65). Insufficient saliva secretion disrupts the balance of oral microbiota and triggers recurrent mucosal inflammation and periodontal lesions. Patients tend to consume soft food persistently to relieve oral pain, which leads to disuse atrophy of masticatory muscles, gradually resulting in weakened chewing strength and delayed swallowing reflexes, namely oral frailty (66). Xerostomia is a significant problem in patients with advanced cancer, yet this symptom is vastly underrecognized and undertreated in routine oncology care (67). A vicious cycle exists between persistent xerostomia and progressive oral frailty.

### Clinical practice

This study provides meaningful clinical implications for refining oral health management strategies in older patients with cancer. First, routine oral frailty screening via validated instruments including the OFI-8 should be integrated into standardized admission evaluation protocols for this high-risk sub-population. Close clinical monitoring is warranted for patients with denture dependency, malnutrition, xerostomia, or inadequate oral hygiene, as these individuals are susceptible to progressive oral functional deterioration. Second, the confirmed robust correlation between denture wearing and oral frailty highlights the necessity of regular prosthodontic assessment and timely oral functional rehabilitation for older patients with cancer. Third, distinct sex-specific discrepancies in oral frailty correlates observed in this study support the adoption of sex-stratified intervention regimens. Individualized oral health management protocols should fully incorporate gender-based differences to optimize targeted clinical efficacy (68). Interdisciplinary collaboration among stomatologists, oncologists, geriatric physicians, and nursing staff is indispensable throughout the entire oncological treatment course. Accumulated evidence has demonstrated that continuous dental involvement in cancer care alleviates oral and systemic adverse complications in cancer survivors (69). In addition, telemedicine-assisted oral health surveillance may serve as a feasible and scalable modality to achieve standardized and uninterrupted oral care management. Early detection of subclinical oral dysfunction and implementation of evidence-based targeted interventions may interrupt the bidirectional pathogenic cycle among oral functional impairment, nutritional deficiency, and physical frailty. Collectively, these integrated clinical strategies also may improve comprehensive oncological prognosis and sustain long-term quality of life in older patients with cancer.

### Limitations

Several limitations of this study should be acknowledged. First, convenience sampling may have introduced selection bias. All participants were recruited through voluntary enrollment at a single hospital, and this sampling approach may induce selection bias and the Hawthorne effect, whereby participants' self-reported behaviors and symptoms are altered by awareness of research participation (70). Voluntary enrollment further compromises sample representativeness, as individuals who are willing to participate and hold greater interest in the study topic are more likely to be included. Second, the cross-sectional design prohibits causal inference between oral frailty, xerostomia, denture, nutritional Status and oral health in older patients with cancer, as temporal trajectories of the investigated variables could not be captured in the current study. Future prospective cohort studies are therefore warranted to collect detailed treatment data, enlarge sample size, and conduct external validation in independent multicenter populations to establish robust causal evidence. Third, xerostomia was evaluated via a single self-reported item instead of validated standardized questionnaires, and this simplified measurement method may reduce the accuracy and reliability of xerostomia assessment. Fourth, the relatively low Cronbach's alpha values reflected weak internal consistency of the study scales. Suboptimal internal consistency may attenuate statistical correlations, produce unstable scale scores, and impede result interpretation, and random measurement errors may further underestimate correlation and regression coefficients, masking true associations between variables or yielding statistically non-significant findings (71). Fifth, although multicollinearity was quantitatively assessed via variance inflation factor (VIF) analysis, with all VIF values within the acceptable threshold, several observed effect sizes still presented wide confidence intervals. This phenomenon reflects low statistical precision and may limit the robustness and generalizability of the estimated associations. Such wide interval ranges imply considerable uncertainty in the magnitude of the investigated relationships, highlighting the need for larger sample sizes in future analyses to narrow confidence intervals and consolidate statistical reliability. Sixth, while the standard OHAT protocol requires professional on-site oral examination, this study adopted questionnaire-based assessment without formal examiner training and standardized operational procedures. Neither intra-rater nor inter-rater reliability was evaluated, and information on examination equipment and assessment conditions was not documented. These shortcomings may introduce observer bias and measurement inconsistency, potentially compromising the reliability of oral health assessment results. Finally, several clinically relevant confounding factors were not collected in this study, including treatment modalities (chemotherapy, radiotherapy, surgery, and immunotherapy), cancer pain, opioid and xerogenic medication use, ECOG performance status, BMI, functional dependence, and cognitive impairment. Unmeasured residual confounding may distort the observed associations, and future multicenter longitudinal studies are needed to incorporate comprehensive clinical confounders, mitigate single-center sampling limitations, and clarify the underlying mechanisms linking oral frailty and dysphagia among older patients with cancer.

## Conclusions

Oral frailty was common among older cancer patients. Denture and malnutrition may help identify patients at increased likelihood of oral frailty. Therefore, early identify and appropriate treatment were required given the differences in the associated factors to oral frailty among men and women older cancer patients.

## Data Availability

The raw data supporting the conclusions of this article will be made available by the authors, without undue reservation.
